# Effects of Bakuchiol on chondrocyte proliferation via the PI3K‐Akt and ERK1/2 pathways mediated by the estrogen receptor for promotion of the regeneration of knee articular cartilage defects

**DOI:** 10.1111/cpr.12666

**Published:** 2019-08-12

**Authors:** Kang Xu, Yongqiang Sha, Sixiang Wang, Qingjia Chi, Yanju Liu, Chunli Wang, Li Yang

**Affiliations:** ^1^ National Innovation and Attracting Talents “111” base, Key Laboratory of Biorheological Science and Technology, Ministry of Education, College of Bioengineering Chongqing University Chongqing China; ^2^ Hubei Engineering Technology Research Center of TCM Processing, College of Pharmacy Hubei University of Chinese Medicine Wuhan China; ^3^ Department of Cardiovascular Surgery, Tongji Medical College Union Hospital, Huazhong University of Science and Technology Wuhan China; ^4^ Center for Precision Medicine, School of Medicine and School of Biomedical Sciences Huaqiao University Xiamen China; ^5^ Department of Mechanics and Engineering Structure, Hubei Key Laboratory of Theory and Application of Advanced Materials Mechanics Wuhan University of Technology Wuhan China

**Keywords:** cartilage injury, estrogen receptor, *Fructus psoraleae*, molecular docking, RNA‐sequencing

## Abstract

**Objectives:**

Cartilaginous tissue degradation occurs because of the lack of survival of chondrocytes. Here, we ascertained whether bakuchiol (BAK) has the capability of activating chondrocyte proliferation.

**Materials and methods:**

The effect of BAK on the proliferation of rat chondrocytes at a concentration of 10 and 20 µmol/L was investigated. The molecular mechanisms involving target binding and signalling pathways were elucidated by RNA‐sequencing, qPCR, molecular docking and Western blotting. Matrigel mixed with bakuchiol was implanted locally into rat knee articular cartilage defects to verify the activation of chondrocytes due to bakuchiol in vivo.

**Results:**

Bakuchiol implantation resulted in the activation of rat chondrocyte proliferation in a dose‐dependent manner. RNA‐sequencing revealed 107 differentially expressed genes (DEGs) with 75 that were up‐regulated and 32 that were down‐regulated, indicating increased activation of the PI3K‐Akt and cell cycle pathways. Activation of the phosphorylation of Akt, ERK1/2 and their inhibitors blocked the proliferative effect of bakuchiol treatment, confirming its direct involvement in these signal transduction pathways. Molecular docking and siRNA silencing revealed that estrogen receptor‐α (ERα) was the target of bakuchiol in terms of its cell proliferative effect via PI3K activation. Two weeks after implantation of bakuchiol, the appearance and physiological structure of the articular cartilage was more integrated with abundant chondrocytes and cartilage matrix compared to that of the control.

**Conclusions:**

Bakuchiol demonstrated significant bioactivity towards chondrocyte proliferation via the PI3K‐Akt and ERK1/2 pathways mediated by estrogen receptor activation and exhibited enhanced promotion of the remodelling of injured cartilage.

## 1 INTRODUCTION

Dietary supplements and traditional herbal medicines are considered to be forms of complementary and alternative medicine that are extensively used globally and widely used for the prevention of cartilage degeneration in Asia and other developed countries. It has been demonstrated that *Fructus psoraleae* (*FP*), the seeds of *Psoralea corylifolia* L. (Leguminosae), exhibits powerful effects against diseases of the skeletal system.^1^, ^2^, ^3^ It has been established that bakuchiol, a natural meroterpenoid compound extracted from *Fructus psoraleae*, exhibits antioxidant,^4^ antitumour,^5^ antimicrobial^6^ and anti‐inflammatory activity.^7^

We have previously demonstrated that components of extracts of *fructus psoraleae* demonstrate the potential to treat cartilage degeneration induced by chondrocyte death.^8^ In that study, we found that bakuchiol was present at a concentration as high as 84.5% of the active extract and that it was the principal cartilage protective component within the extract. This discovery has motivated us to continue to explore the therapeutic possibilities of bakuchiol against cartilage diseases.

Osteoporosis (OP) and osteoarthritis (OA) are high‐risk diseases of the elderly, whose main features are degeneration of the cartilaginous matrix and apoptosis of functional chondrocytes.^9^ In addition to anti‐inflammatory treatments, it is more important in OP and OA to activate autologous chondrocytes to proliferate and produce cartilaginous extracellular matrix so as to replenish the degenerated matrix.^10^ Therefore, promotion of chondrocyte growth is a key factor in the treatment of that form of cartilage damage. Lim et al compared different compounds from *Psoralea corylifolia* L. and demonstrated that bakuchiol is a key component with oestrogenic activity due to high estrogen receptor‐binding affinity.^11^ Several studies have confirmed that oestrogen and similar active compounds regulate the cell cycle and therefore proliferation and growth.^12^, ^13^ These findings provide the basis for the hypothesis that bakuchiol, as a compound with oestrogenic activity, has a potential role as a therapy of degenerative diseases of cartilage.

For the first time, in this study and in accordance with our initial hypotheses and experimental results, we have demonstrated that bakuchiol, a bioactive natural product, caused significant stimulation of chondrocyte proliferation via the PI3K‐Akt and ERK1/2 signal transduction pathways mediated by estrogen receptors. It also usefully exhibited promotion of injured cartilage remodelling. The addition of BAK to medications and food supplements has the potential to treat cartilage degeneration and osteoarthritis.

## 2 MATERIALS AND METHODS

### 2.1 Cell culture and treatment

Rat chondrocytes were isolated using a method adapted from a previous study.^9^ Briefly, articular cartilage was retrieved from the knees of healthy Sprague‐Dawley rats (male, n = 5, weight: 150 g). The cartilage was minced, and pieces digested with 2 mg/mL of collagenase type II (Gibco) for 2 hours. After removal of undigested tissue, the cells were cultured in DMEM/F12 (1:1) supplemented with 10% FBS. Passage 2‐3 (P2‐3) cells were used in the experimental studies. The bakuchiol was purchased from Selleck (S3813). Animal experiment protocol was reviewed and approved by the Institutional Review Board (IRB) of Chongqing University.

### 2.2 Cell viability testing

Cell viability in response to BAK was evaluated using a cell counting kit‐8 (CCK8) assay which was also utilized to assess cell viability. Cells were seeded at a density of 1 × 10^4^ cells/well in a 24‐well plate and the cytotoxicity of BAK tested using concentrations of 1 to 100 μmol/L for 3 days. The cells were then treated with 10 μmol/L BAK for 7 days. The cells were then washed with PBS and incubated in serum‐free medium containing 10% CCK8 reagent for 2‐3 hours at 37°C in an atmosphere containing 5% CO_2_. Aliquots were pipetted into a 96‐well plate and absorbance at 490 nm measured using a plate reader (Bio‐Rad).

### 2.3 Cell proliferation assay

Chondrocyte proliferation was quantified using an ethynyl deoxyuridine (EdU) DNA in vitro proliferation detection kit (Guangzhou RiboBio) using flow cytometry, in accordance with the manufacturer's instructions.^14^ Images of cells were captured by light with microscopy using Image‐Pro Plus 6.0 software (Media Cybernetics). The number of EdU‐labelled cells was calculated from fields randomly selected in each well. Numbers of cells in each phase of the cell cycle were calculated using a BD FACS Calibur Flow Cytometer at a 488 nm excitation wavelength.

### 2.4 qRT‐PCR

Cells were harvested using Trizol reagent (Invitrogen), followed by RNA isolation. Each sample was reverse transcribed to cDNA using a RevertAid First Strand cDNA Synthesis Kit (Thermo Fisher Scientific). The reverse transcription product was then used as a template to perform real‐time polymerase chain reaction (PCR) on a StepOne Plus thermal cycler (Applied Biosystems) using PowerUp™ SYBR™ Green Master Mix (Applied Biosystems), following the manufacturer's guidelines. The primers for CDK1 (F: 5′‐TCCTCCAGGGGATTGTGTTTT‐3′; R: 5′‐GCCAGTTTGATTGTTCCTTTGTC‐3′), E2F1 (F: 5′‐ACTTTGGTCTCGAGGAGGGT‐3′; 5′‐TGCTATTCCAACGAGGCAGG‐3′), MKI67 (F: 5′‐GCCCCTGGAAGATTATGGTGG‐3′; R: 5′‐GGGTTCTGACTGGTTGTGGTTGT‐3′) were synthesized by Invitrogen. The final data were analysed by the 2^−ΔΔ^ *^C^* ^t^ method.

### 2.5 Western blotting

Western blotting analysis was performed as described previously. After separation using SDS‐PAGE then transfer to PVDF membranes, the target proteins were detected after incubating with primary antibodies for CDK1 (Abcam: ab18, 1:1000), E2F1 (Abcam: ab179445, 1:1000), phosphorylated Akt (CST: 9271, 1:1000), Akt (CST: 9272, 1:1000), phosphorylated ERK1/2 (Abcam: ab201015, 1:1000), ERK1/2 (Abcam: ab36991, 1:1000) and ERα (Santa Cruz: sc‐8005, 1:200). The membranes were then incubated with the corresponding secondary antibody at 37°C for 1 hour Finally, the immunoreactive bands were developed using SuperSignal West Femto Maximum sensitivity substrate (Thermo Fisher Scientific), and the images analysed using Quantity One Software (Bio‐Rad).

### 2.6 RNA‐sequencing for detection of DEGs and pathways

RNA‐sequencing (RNA‐seq) analysis and quantification were utilized to investigate changes in cell mRNA profiles among the different treatments performed. Isolated RNA was sent to BGI Co., Ltd. for conducting RNA‐seq, which was performed on a BGISEQ‐500 (Shenzhen, China). All samples were replicated three times for confirmation purposes. Sequencing results were further analysed using R programming language and software (version 3.5.1), in order to identify differentially expressed genes (DEGs), and perform Gene Ontology (GO) and Kyoto Encyclopedia of Genes and Genomes (KEGG) pathway enrichment analysis.

### 2.7 Target prediction and molecular docking

The online tool “SwissTargetPrediction” was used to predict potential targets of BAK compounds.^15^ Simplified molecular input line entry specification (SMILES) was used and the molecule identified within the NCBI PubChem Compound database. Molecular docking analysis was performed using Autodock 4^16^ and protein information identified using the RCSB PDB protein data bank.

### 2.8 Evaluation of the effect of BAK on PI3K‐Akt and ERK pathways via estrogen receptor activation

The PI3K‐Akt and ERK signalling pathways were involved in the regulation of cell proliferation by BAK. Two methods of analysis were conducted to verify this. Firstly, the cells were treated with BAK or BAK plus the Akt or ERK inhibitors PI‐103 and SCH772984, respectively. Following culture of the cells, Western blotting was used to ascertain if Akt or ERK1/2 were phosphorylated and to verify the association between BAK and the activation of the Akt and ERK1/2 pathways. Secondly, ERα siRNA (ERα si‐R) was used to knockdown estrogen receptor in the target cells, which were then further cultured for 48 hours with BAK. Meanwhile, the non‐treatment group was used as the control. Finally, the protein expression levels of CDK1 and phosphorylated and total Akt (p/t‐Akt) were detected using Western blotting so as to confirm the involvement of the ERα/PI3K‐Akt/ERK axis signalling pathways in cell proliferation by BAK stimulation.

### 2.9 Animal experiments

Animal experiments were conducted in accordance with protocols approved by the Chongqing University Animal Care and Use Committee. For the cartilage injury model, a 0.5‐mm‐deep thickness defect was created through the articular cartilage and subchondral bone of the patellar groove of Sprague‐Dawley (SD) rats (200‐250 g) using a micro‐electrical drill. Matrigel mixed with bakuchiol (100 µmol/L) was implanted locally to fill the cartilage defect. Articular cartilage samples were harvested after 1 and 2 weeks post‐implantation for macroscopic and histological evaluation, with analysis by micro‐CT.

### 2.10 Histological analysis

The harvested samples were embedded in paraffin, sectioned for 10 μm thickness and then stained with haematoxylin and eosin (H&E), Safranin‐O green and Alcian blue. The sections were scored histologically in accordance with previous studies.^9^, ^17^, ^18^ The relative area of each defect (Figure 6: area within the dashed line) in the different groups was measured using Image J. The relative content of cartilage matrix was ascertained from the degree of Safranin staining (Red area) and GAG content by Alcian blue staining (Positive blue area). All morphological measurements were performed using Image J. The mean histological scores for each group were statistically analysed, with t tests performed between the BAK‐treated group and control. In addition, the histological immunofluorescent staining for Ki67, p‐Akt, and p‐ERK was performed to verify the in vivo mechanisms of BAK on cell proliferation.

### 2.11 Statistical analysis

RNA‐sequencing results were further analysed using R programming language and software (version 3.5.1). All other data were analysed and expressed as means ± standard deviation (SD). Molecular docking was analysed using Autodock 4. Differences among groups were determined statistically using analysis of variance (ANOVA). A *P* value <.05 was considered statistically significant.

## 3 RESULTS

### 3.1 Bakuchiol stimulates chondrocyte proliferation in vitro

**Figure 1:**
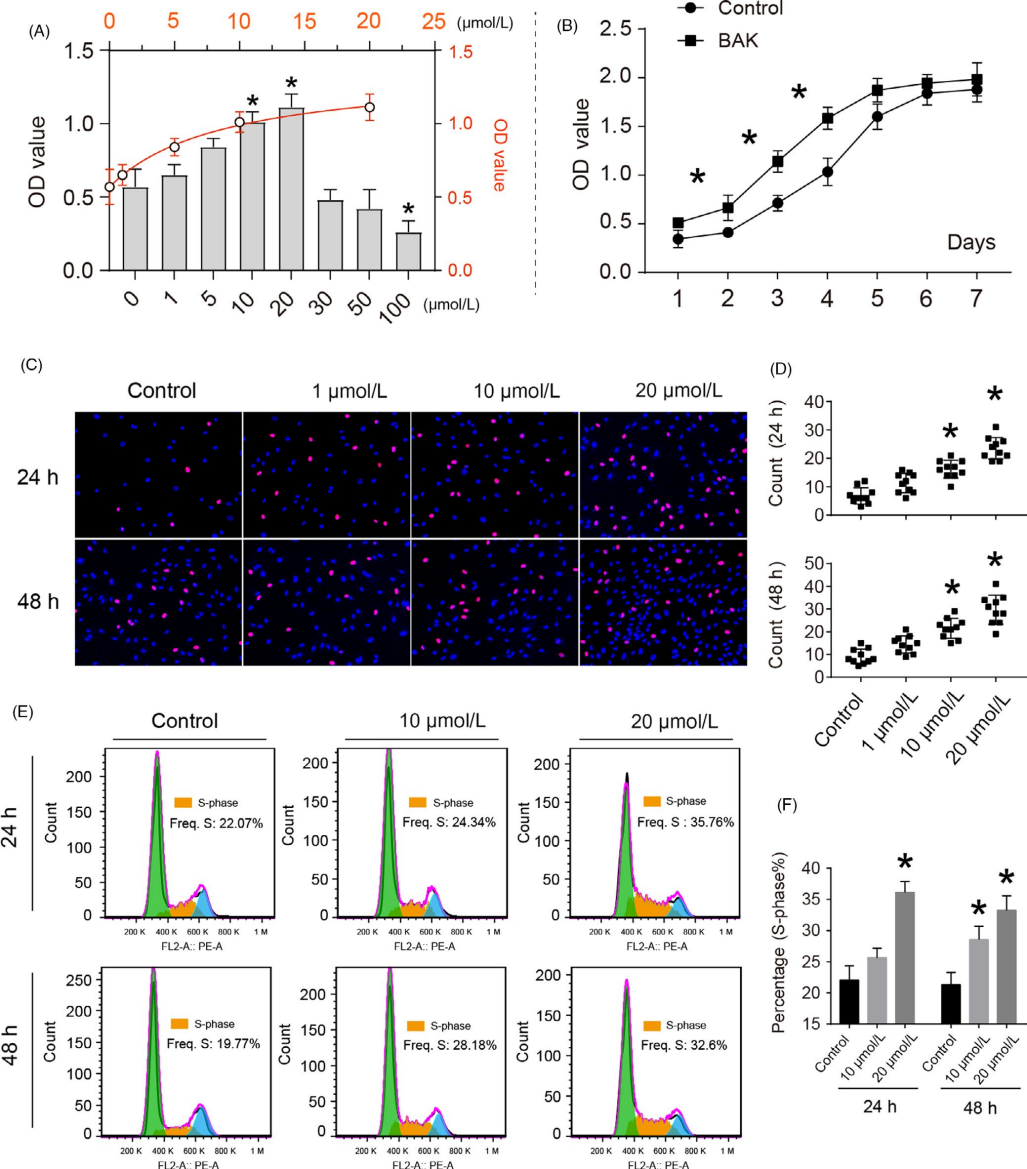
The effects of bakuchiol (BAK) on cell proliferation of chondrocytes. (A) Cytotoxicity testing of BAK at the concentration ranging from 1 to 100 µmol/L for 24 h. (B) Cell proliferation curve with BAK treatment at 10 µmol/L and control for 7 d. (C) Fluorescent staining of EdU assay for detecting DNA synthesis indicating cell proliferation, (D) Percentage of EdU (red) positive staining statistical analysis, the data are presented as mean ± SD (n = 10, random fields), (*) *P* < .05 (vs control) were accepted as statistically significant. (E) Flow cytometry for cell cycle analysis of chondrocytes with BAK treatment (10 and 20 µmol/L) for 24 and 48 h, (F) S‐phase was counted and made statistical comparison. * *P* < .05 (vs control) was accepted as significant difference, n = 3

Results of the CCK8 assay indicated that no cytotoxic effects of exogenously applied BAK (1 to 100 µmol/L) were observed on chondrocytes after 24 hours. By contrast, 100 µmol/L BAK exhibited clear toxicity in the cells (*P < .05) (IC50: 48.545 µmol/L; Figure S1). The EC50 of BAK was found to be 8.897 µmol/L (Figure 1A). Concentrations of 5, 10 and 20 µmol/L increased proliferation significantly (*P < .05) for chondrocytes vs the control group (0.1% DMSO; Figure 1A). After 7 days of culture, supplementation with 10 µmol/L BAK promoted cell viability in comparison with the control group (*P < .05; Figure 1B). Fluorescent staining of EdU in the proliferation assay indicated that 10 and 20 µmol/L BAK treatments significantly promoted cell proliferation after 24 and 48 hours (Figure 1C and 1). Notably, the numbers of cells involved in genome replication (S‐phase) were clearly increased when chondrocytes were incubated in 10 and 20 µM BAK‐conditioned culture medium after 24 and 48 hours (*P < .05; Figure 1E,F).

### 3.2 Gene expression profiles reveal differentially expressed genes and enriched pathways

**Figure 2:**
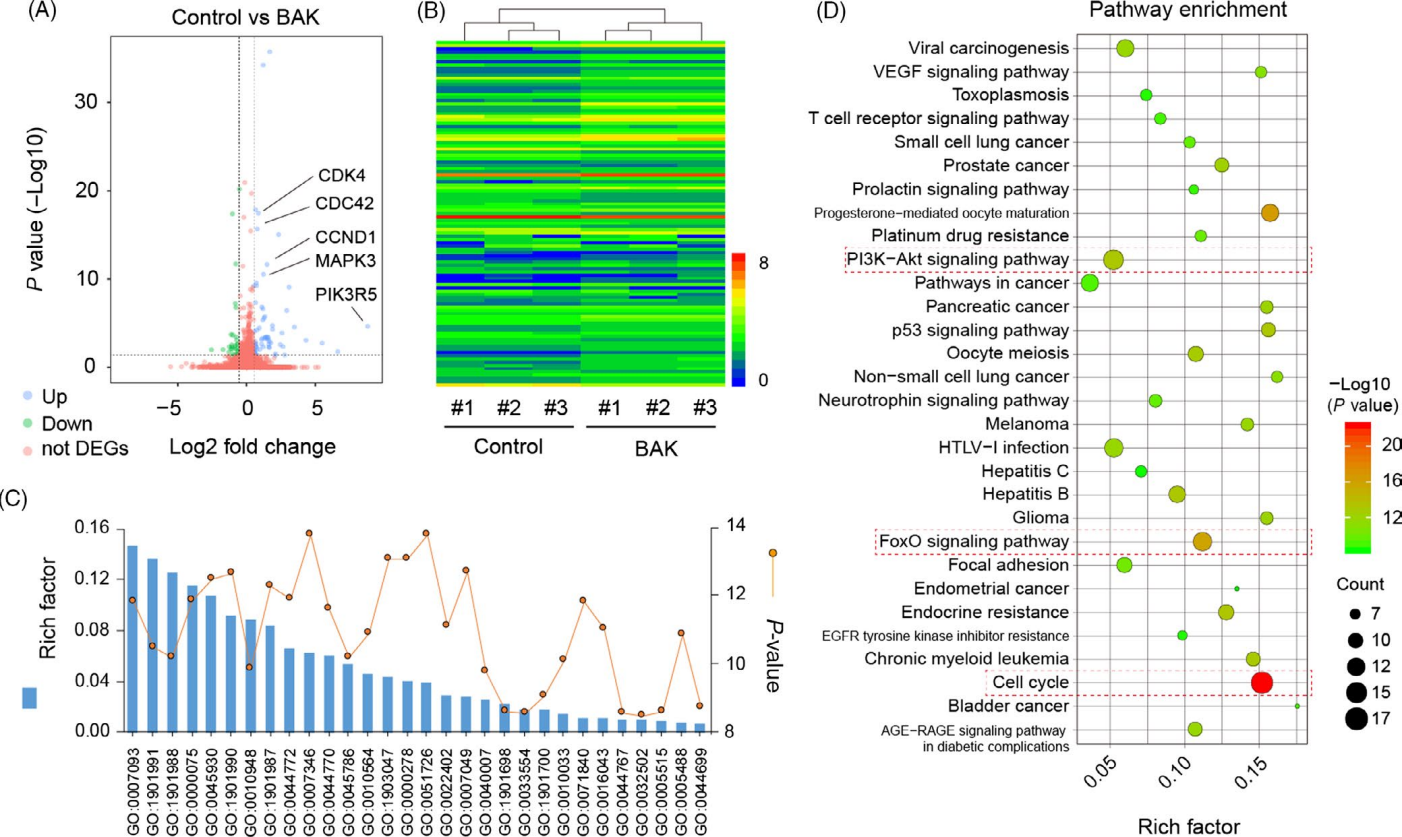
Global gene expression profiles with or without 10 µmol/L BAK treatment. (A) Volcano map of differentially expressed genes (DEGs) in BAK vs control (up‐regulation: 75 and down‐regulation: 32), FC (fold change)> 1 was accepted as positive DEGs. (B) Heatmap for global gene expression with group clusters (n = 3), (C) GO enrichment of those DEGs, line indicates *P*‐value (−log10). (D) Pathway enrichment bubble map based on the KEGG enrichment analysis, a larger *P* value (−Log10) indicates a higher degree of enrichment

The RNA‐seq experiments demonstrated that a significant number of genes in chondrocytes, when treated with 10 µmol/L BAK, were differentially expressed compared with control cells, with 75 genes that were up‐regulated and 32 down‐regulated (Figure 2A). Additionally, global gene expression profiles demonstrated that large differences in gene expression regulation occurred following the addition of BAK (Figure 2B), compared with the control group. Based on the analysis of DEGs and the GO functional annotations described above, KEGG signal pathway enrichment analysis was performed on the DEGs that were identified (Figure 2C). Our results demonstrated that these DEGs were highly enriched in functions related to PI3K‐Akt, FoxO and cell cycle signalling pathways, among others (Figure 2D).

### 3.3 Bakuchiol regulates proliferative gene/protein expression

**Figure 3:**
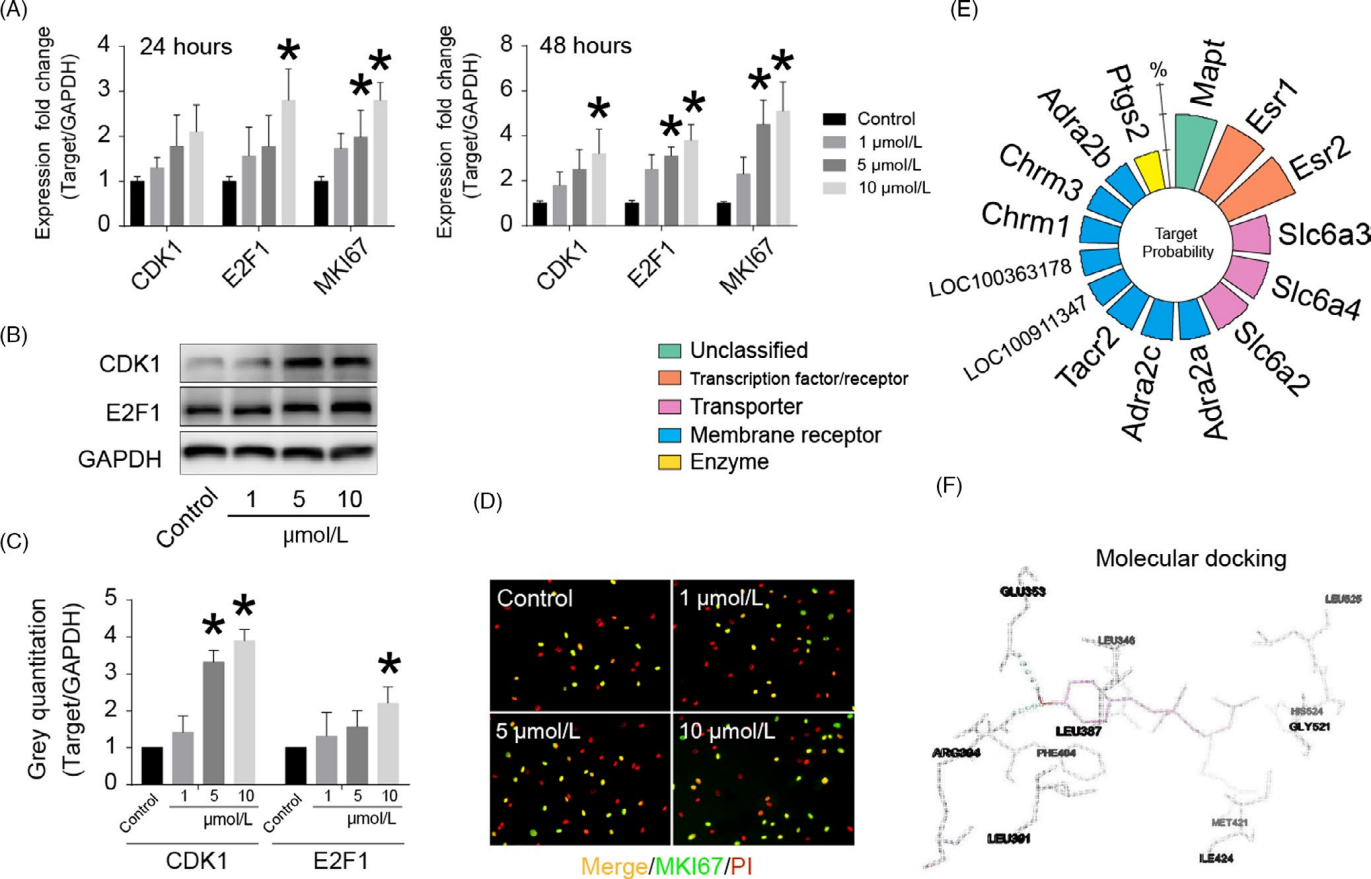
BAK up‐regulates proliferative gene/protein expression and the molecular targets prediction and docking of BAK. (A) The effects of BAK (1 to 10 µmol/L) on gene expression of CDK1, E2F1, and MKI67 for 24 and 48 h. (B) The effects of BAK (1 to 10 µmol/L) on protein synthesis of CDK1 and E2F1 for 3 d, (C) Statistical analysis of CDK1 and E2F1 according to (B), the data are collected by grey semi‐quantification, and presented ratio to GAPDH, (*) *P* < .05 (vs control) was accepted as statistically significant. (D) Immunofluorescent staining of MKI67. (E) Nightingale roses from the online tool “SwissTargetPrediction,” percentage indicates probabilities in the top 15 targets. (F) Esr1 was selected for molecular docking analysis, BAK successfully accessed the pocket structure of the protein molecule: Esr1 with residue: GLU353 and ARG394 (green dashline) for hydrogen bond interaction and others

Chondrocytes were treated with BAK (1 to 10 µmol/L) for 24 and 48 hours. Compared with the control, BAK was found to activate chondrocytes and induce cell proliferative gene expression and protein synthesis in chondrocytes (Figure 3A). 10 µmol/L BAK resulted in significantly up‐regulated mRNA levels of CDK1 (*P < .05), E2F1 (*P < .05) and MKI67 (*P < .05) after 48 hours. Similar to the gene expression results described above, BAK not only stimulated CDK1 protein synthesis but also up‐regulated E2F1 at the translation level (Figure 3B and 3). According to immunofluorescent staining (Figure 3D), 10 µmol/L BAK clearly promoted MKI67 protein synthesis (*P < .05) relative to the control. SwissTargetPrediction provided suggestions of more than 15 potential molecular targets for BAK (Figure 3E). When combined with a literature analysis of BAK, Esr1 was selected for further molecular docking analysis (Figure 3F). The results demonstrated that BAK can successfully bind to Esr1 using the residues GLU353 and ARG394 (green dashed line) via hydrogen bond interactions and residues LEU391, PHE404, LEU387, LEU346, MET421, ILE424, LEU525 and GLY521.

### 3.4 Bakuchiol promotes cell proliferation via PI3K‐Akt and ERK1/2 pathways mediated by ERα

**Figure 4:**
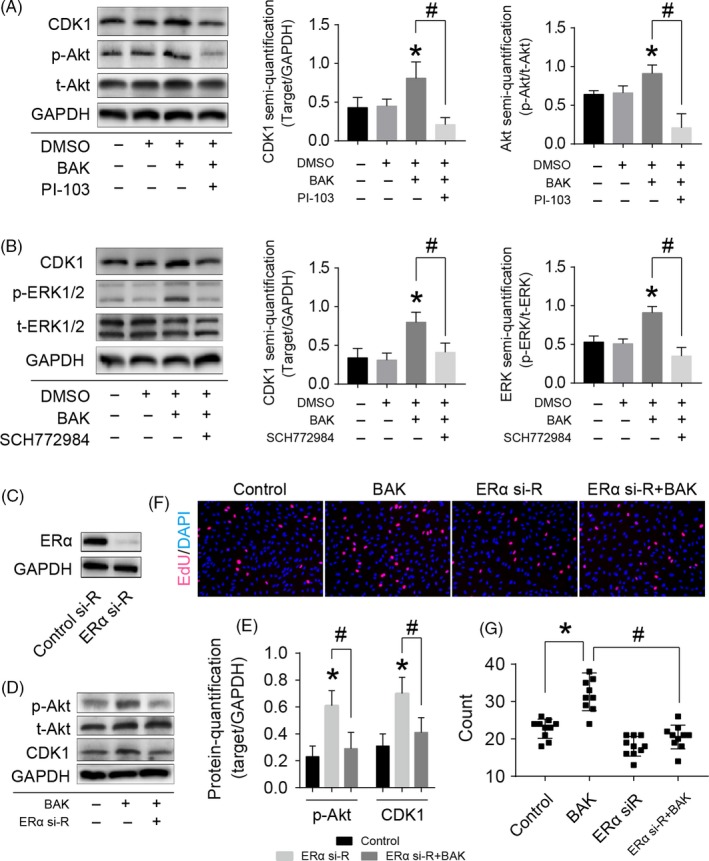
BAK promotes cell proliferation via PI3K‐Akt and ERK1/2 pathways mediated by ERα. (A) Western blotting for CDK1, phosphorylated Akt (p‐Akt), and total Akt (t‐Akt) of the cells with 10 µmol/L BAK and 1 µmol/L PI‐103 (PI3K inhibitor) treatment, statistical analysis of CDK1 and Akt were collected according to the grey semi‐quantification and presented ratio to GAPDH, (*) *P* < .05 (vs control) and (#) *P* < .05 was accepted as statistically significant. (B) Western blotting for CDK1, phosphorylated ERK1/2 (p‐ERK1/2), and total ERK1/2 (t‐ERK1/2) of the cells with 10 µmol/L BAK and 1 µmol/L SCH772984 (ERK1/2 inhibitor) treatment, statistical analysis of CDK1 and ERK1/2 collected according to the grey semi‐quantification and presented ratio to GAPDH, (*) *P* < .05 (vs control) and (#) *P* < .05 was accepted as statistically significant. (C) Western blotting for detecting ERα expression to verify ERα si‐RNA (ERα si‐R) knockdown efficiency for 72 h. (D) Western blotting for detecting p‐Akt, CDK1 with BAK plus ERα knockdown, (E) the grey intensity was measured, (*) *P* < .05 (vs control) and (#) *P* < .05 were accepted as statistically significant. (F and G) EdU assay for verifying cell proliferation effect with BAK plus ERα knockdown, (*) *P* < .05 and (#) *P* < .05 were accepted as statistically significant, n = 10 (random fields)

The results demonstrated that BAK significantly stimulated CDK1 protein synthesis and improved the ratios of phosphorylated‐/total Akt (p/t‐Akt) and phosphorylated‐/total‐ERK1/2 (p/t‐ERK1/2; Figure 4). Furthermore, CDK1 protein levels and the p/t‐Akt ratio in chondrocytes reduced when cultures were supplemented with PI‐103, suggesting that the PI3K‐Akt signalling pathway was involved in BAK’s regulation of CDK1 expression in chondrocytes (Figure 4A). Similarly, CDK1 protein levels and p/t‐ERK1/2 ratios in chondrocytes reduced following supplementation of cultures with SCH772984, suggesting that the MEK‐ERK1/2 signalling pathway also interfered in BAK’s regulation of CDK1 expression in chondrocytes (Figure 4B). The efficiency of the ERα siRNA designed to silence the estrogen receptor gene was measured in chondrocytes (Figure 4C). Chondrocytes were incubated with ERα‐siRNA (ERα si‐R) and then treated with BAK for 48 hours. Treatment with BAK increased CDK1 protein expression and DNA synthesis in chondrocytes; however, ERα‐siRNA antagonized these effects (Figure 4D‐G). In comparison with the BAK group, the phosphorylation of Akt declined after ERα‐siRNA supplementation (#*P* < .05; Figure 4E). This indicates that BAK‐induced chondrocyte proliferation through the PI3K‐Akt and ERK1/2 pathways is mediated by ERα.

### 3.5 Bakuchiol promotes remodelling of injured knee articular cartilage defects

**Figure 5:**
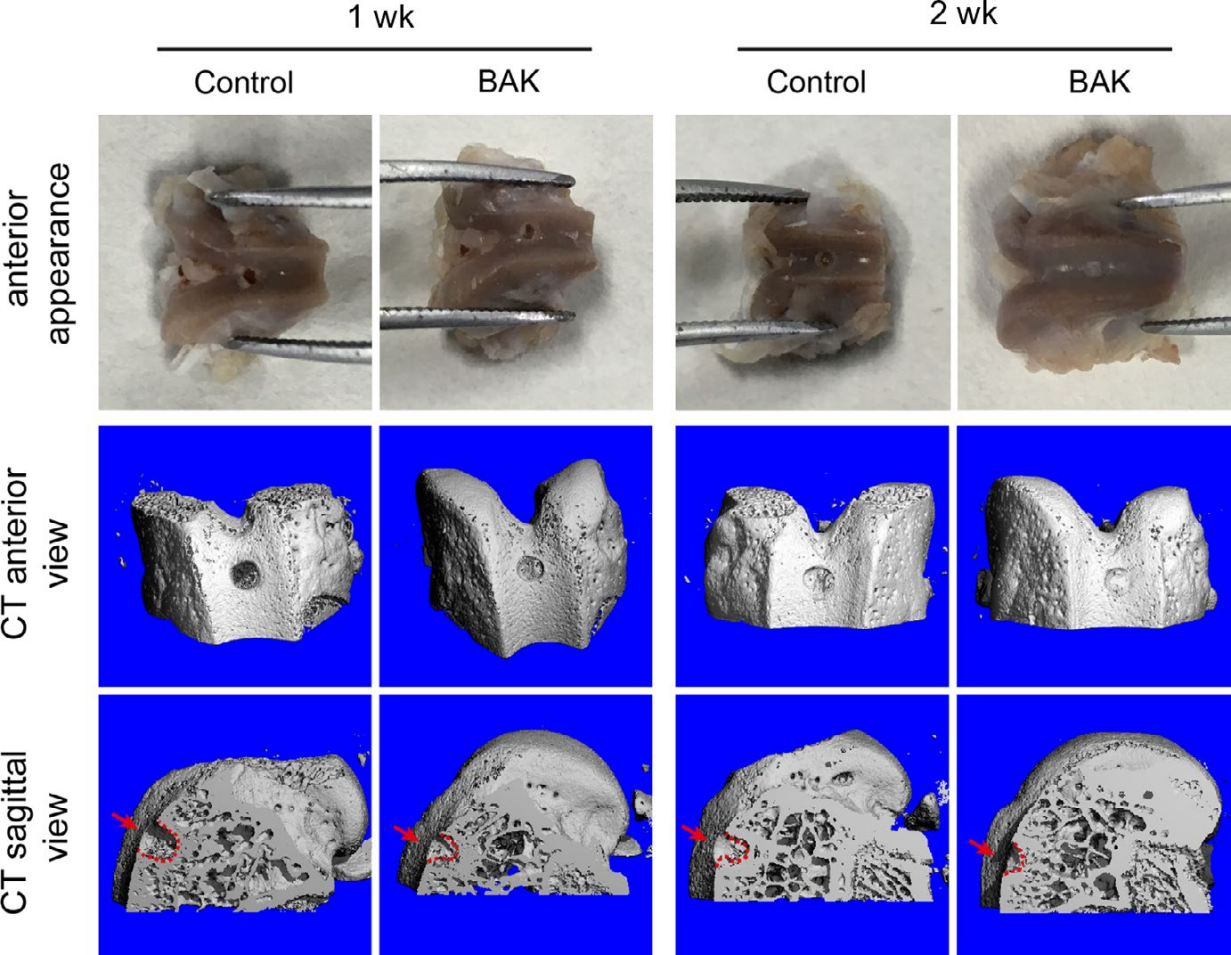
Macroscopical and micro‐CT evaluation on knee articular cartilage of rats with or without BAK treatment for 1 and 2 wk, the defective hole is 0.5 mm deep with micro‐electrical drilling, and arrows and dashline indicate defect site

**Figure 6:**
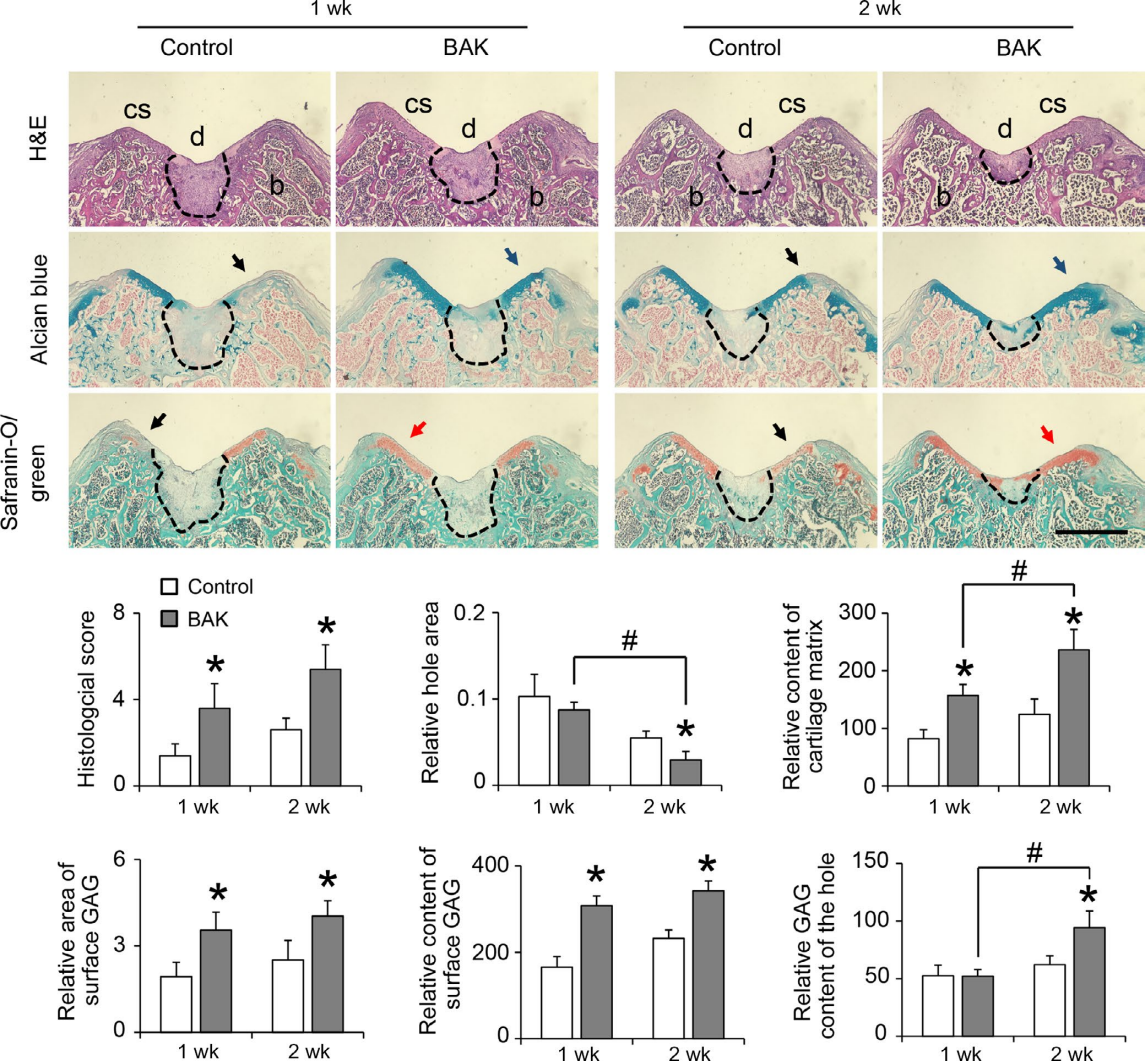
Histological staining of H&E, Alcian blue and Safranin‐O/green for 1 and 2 wk after defective injury on knee articular cartilage of rats with or without BAK treatment, cs: cartilage surface, d: defective injury (by dashline), b: bone. Black arrows indicate degenerated cartilage matrix, blue arrows indicate abundant glycosaminoglycan, and red arrows indicate constructive cartilage matrix. For the statistical analysis, (*) *P* < .05 and (#) *P* < .05 were accepted as statistically significant, bar: 500 µm

After 1 week, the central regions of the defect sites were not filled with cartilage matrix in either the control or BAK group, indicating that cartilage repair remained limited. However, according to the micro‐CT imaging, the patellar fossa was significantly larger in the control group than in the BAK‐treated group at this time point (Figure 5). The defects in the BAK‐treated group were filled with a shiny, smooth, hyaline‐like tissue after 2 weeks compared with the control group (Figure 6). Compared with the controls after 1 and 2 weeks, the histological scores of the BAK‐treated group defects were 3.60 ± 0.54 (*P < .05) and 5.40 ± 0.55 (*P < .05) respectively. It is worth noting that not only were the histological scores significantly different, but so also was the content of GAG, with BAK treatment clearly different (*P < .05) from the control (Figure 6). In addition, with BAK treatment, no significant depression was observed after 2 weeks in comparison with its appearance after 1 week (#*P* < .05), suggesting that the articular cartilage repair was complete. Taken together, these data suggest that BAK protected in situ chondrocyte viability, prevented degradation of the articular cartilage, and provided optimal regeneration of the cartilage and bone defects.

**Figure 7:**
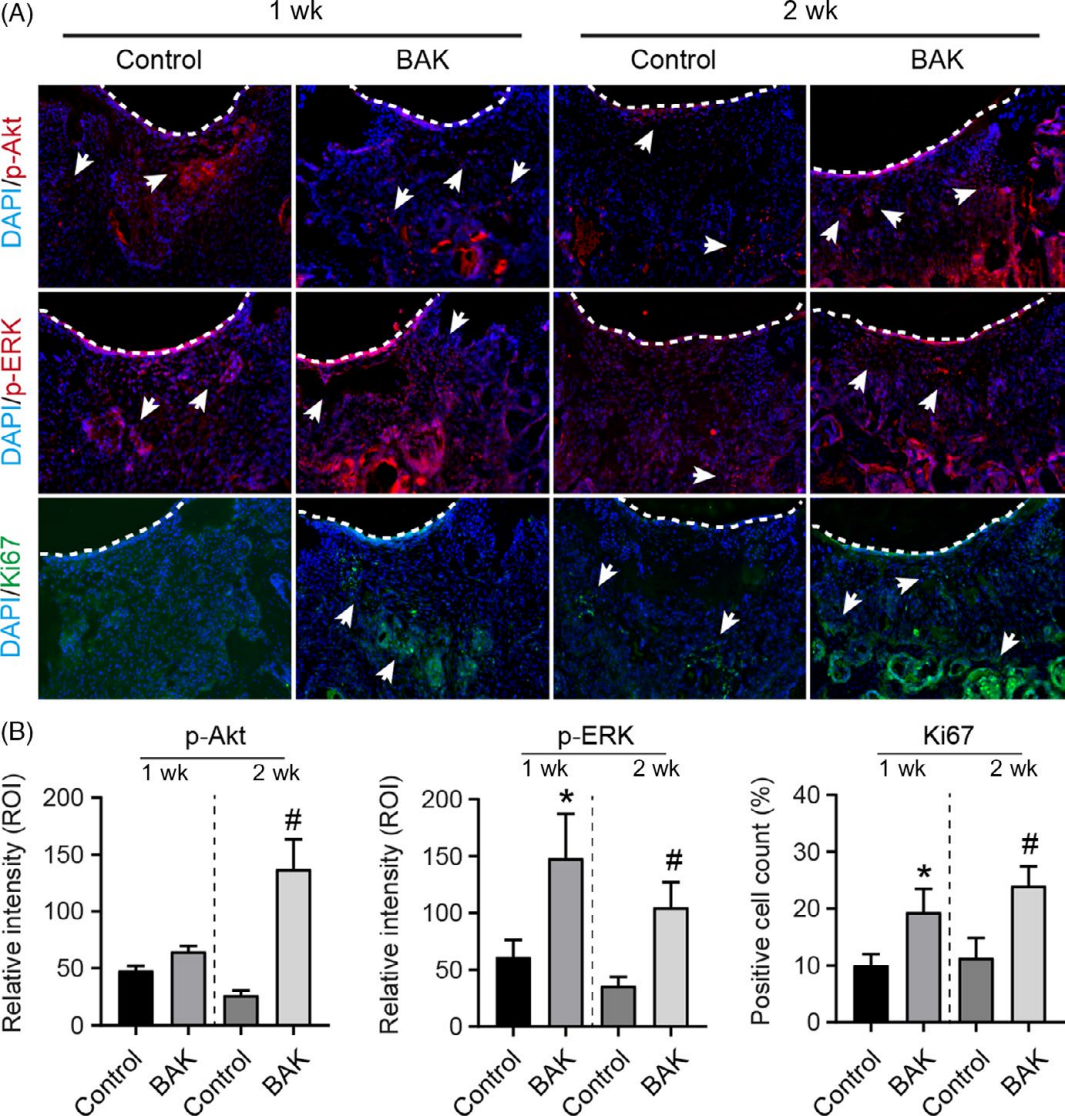
Immunofluorescent staining of p‐Akt, p‐ERK1/2 and MKI67 (Ki67) for 1 and 2 wk after defective injury on knee articular cartilage of rats with or without BAK treatment. A, Dashline indicates cartilage surface of defective injury site, and arrows indicate positive staining. B, Semi‐quantitative analysis, (*) *P* < .05 (vs control in 1 wk) and (#) *P* < .05 (vs control in 2 wk) were accepted as statistically significant

In addition, the expression of Ki67, p‐ERK and p‐Akt was measured in situ in the defect site by immunofluorescent staining (Figure 7). Positive fluorescent intensity (arrows in Figure 7) relating to p‐ERK and p‐Akt was greater following treatment with BAK implantation after both 1 and 2 weeks post‐injury. Semi‐quantitative analysis (Figure 7B) showed significant differences with BAK treatments when compared to control in 1 and 2 weeks (*P < .05 in 1 w; #*P* < .05 in 2 w). In the BAK‐treated group, a greater number of cells in the defect site (below the dashed line in Figure 7) stained positive for Ki67 (Figure 7B, * *P* < .05, vs control in 1 week; # *P* < .05, vs control in 2 week). These results indicate that BAK promoted the expression of Ki67, p‐ERK and p‐Akt in vivo.

## 4 DISCUSSION

In a previous study, we demonstrated that components in extracts of fructus psoraleae demonstrated the potential to treat cartilage degeneration induced by chondrocyte apoptosis. In that study, we found that the percentage content of bakuchiol (BAK) in the active fructus psoraleae extract was as high as 84.5%, as measured by HPLC analysis and the principal active compound in the extract.^8^ Combined with the results of the cell proliferation experiments, we believe that the substance that actively promotes chondrocyte proliferation is bakuchiol. In addition, in a previous study of *FP* extract on chondrocyte proliferation, we found that petroleum ether extracts of *FP* at concentrations greater than 0.1 mg/mL revealed significant toxicity. At first, we suspected that this phenomenon was due to residual petroleum ether, but this was not the case. In this study, we treated chondrocytes directly with different concentrations of BAK, the results indicating that with BAK concentrations >20 μmol/L in the medium, significant cytotoxicity was experienced. This result suggests that the effect of BAK on chondrocytes is dose‐dependent, with high concentrations inhibiting cell growth, whereas appropriate concentrations effectively promoted chondrocyte proliferation. Therefore, our subsequent studies focused on a BAK concentration of 10 μmol/L.

Furthermore, in the animal experiments of this study, we amplified the effective concentration of BAK by a factor of 10, to reach 100 μmol/L. This was mainly because, on the one hand, animal experiments are different from in vitro cell experiments and require a larger quantity of a drug. On the other hand, based on the results of our preliminary experiments, we tested the release profile of BAK in Matrigel (Figure S2), with an in vivo implantation concentration of 100 μmol/L BAK showing therapeutic effects.

We conducted transcriptome high‐throughput sequencing to explore possible signal transduction pathways in order to ascertain the molecular mechanisms for the promotion of proliferation in chondrocytes. The cell cycle, PI3K‐Akt and ERK1/2 pathways were analysed by enrichment of sequencing data. Subsequent studies also confirmed that BAK promoted chondrocyte proliferation and was indeed associated with the pathways described above. However, it was not apparent what the specific target molecule of BAK was. The convenient online tool “SwissTargetPrediction” predicted that the most likely potential target of BAK was the estrogen receptor. Lim et al compared different compounds from Psoralea corylifolia L. and demonstrated that bakuchiol is a key compound with oestrogenic activity due to its high estrogen receptor‐binding affinity.^11^ Several studies have confirmed that oestrogen and similar active compounds regulate the cell cycle and so proliferation.^19^, ^20^, ^21^ Therefore, the hypothesis that “bakuchiol as an active compound demonstrating oestrogenic activity may be used as a therapy for cartilage degenerative diseases” has been proposed.

Based on the in vitro cytology experiments, we further used an in vivo cartilage defect injury model to test the role of BAK in the promotion of cartilage proliferation and repair of damaged cartilage. In addition, we verified that BAK promoted the in vivo expression of Ki67, p‐ERK and p‐Akt in the cells at the defect site. These results demonstrate that BAK protected chondrocyte viability in situ and promoted remodelling of the articular cartilage. Due to the limitations of the experimental conditions, we have no way to conduct mechanistic experiments in vivo on transgenic mice with cartilage‐specific ER knockout. It is impossible to directly confirm that BAK promoted chondrocyte proliferation through ER receptors in vivo. However, the results of the animal experiments presented above suggest that BAK has clearly positive pharmacological effects. Studies have shown that, for cartilage regeneration, in addition to the proliferation of chondrocytes, it is more important to regulate the microenvironment around the cartilage such as inflammation,^22^ angiogenesis 23 or niches appropriate for stem cells.^24^ It has been reported that BAK has certain anti‐inflammatory effects.^25^ For diseases of the cartilage, osteoarthritis is the most common degenerative disease. Its principal pathological feature is a large number of inflammatory reactions.^9^, ^26^ For future cartilage tissue regeneration and repair strategies, the specific mechanism that BAK is capable of promoting that requires additional exploration is inhibition of the inflammatory response.

## CONFLICT OF INTEREST

We declare that the authors have no conflict of interest.

## Data Availability

All data included in this study are available upon request by contact with the corresponding author.
