# Supplementary figures and images for: Effects of Bakuchiol on chondrocyte proliferation via the PI3K‐Akt and ERK1/2 pathways mediated by the estrogen receptor for promotion of the regeneration of knee articular cartilage defects
Source: Cell Prolif. 2019 Aug 12;52(5):e12666. doi: 10.1111/cpr.12666 (PMC6797515; doi:10.1111/cpr.12666)

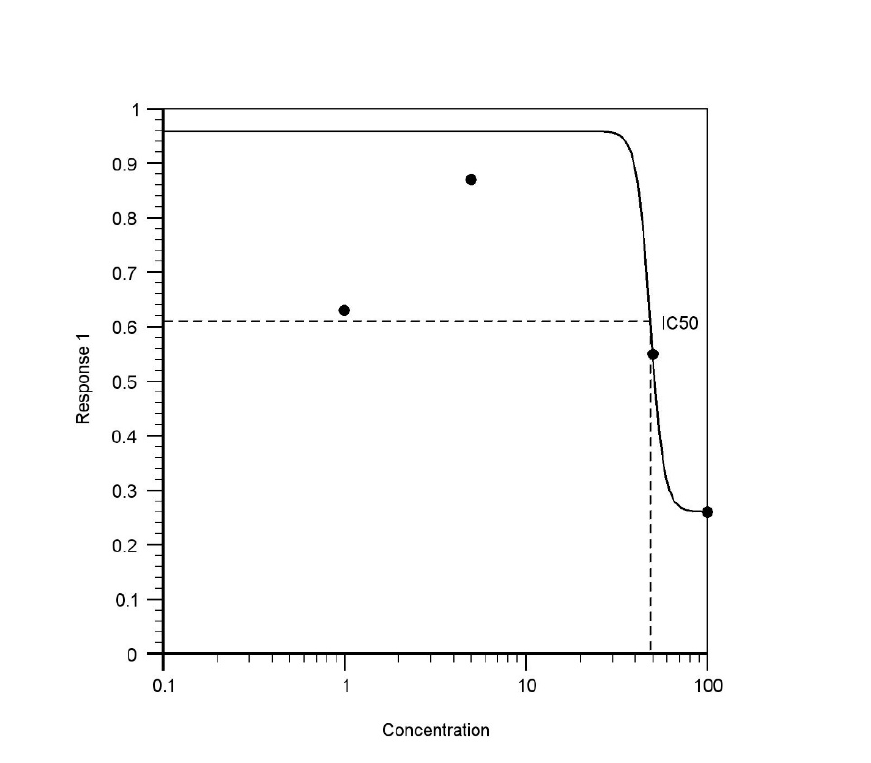

Supplement: Supplementary file 1 [file CPR-52-e12666-s001.tif]

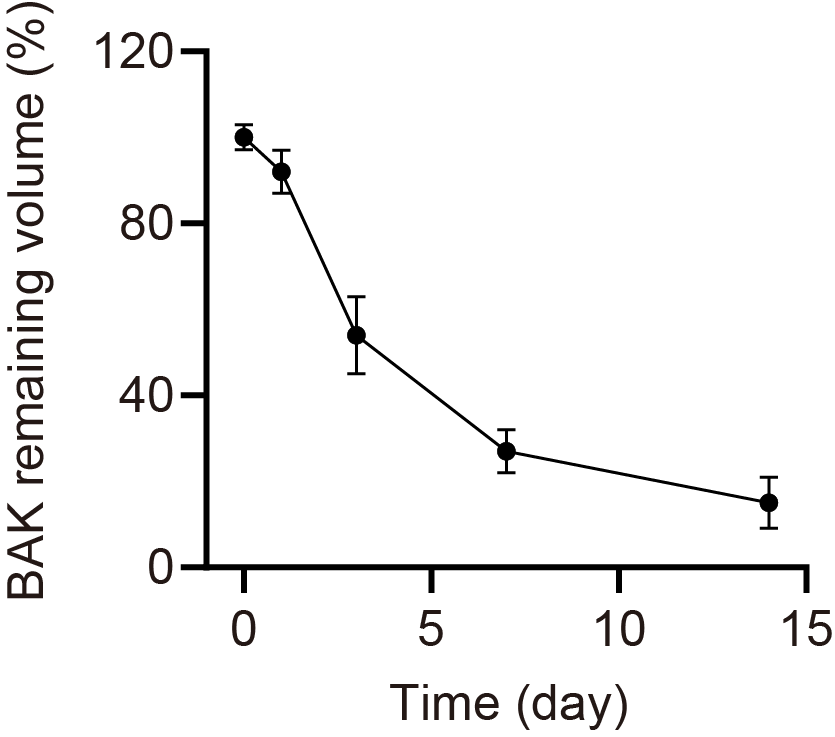

Supplement: Supplementary file 2 [file CPR-52-e12666-s002.tif]
